# Antibodies to *Borrelia burgdorferi* and *Bartonella* species in serum and synovial fluid from people with rheumatic diseases

**DOI:** 10.1128/spectrum.01653-23

**Published:** 2024-03-14

**Authors:** Lisa Kim, Erin Lashnits, Edward B. Breitschwerdt, Amanda Elam, Neenah Grade, Jennifer Miller, Alexander R. Shikhman

**Affiliations:** 1 Department of Medical Sciences, School of Veterinary Medicine, University of Wisconsin-Madison, Madison, Wisconsin, USA; 2 Intracellular Pathogens Research Laboratory and Comparative Medicine Institute, College of Veterinary Medicine, North Carolina State University, Raleigh, North Carolina, USA; 3 Galaxy Diagnostics, Research Triangle, North Carolina, USA; 4 Institute for Specialized Medicine, San Diego, California, USA; Uniwersytet Medyczny w Bialymstoku, Bialystok, Poland

**Keywords:** antibodies, *Bartonella*, *Borrelia burgdorferi*, immunoserology, Lyme, serodiagnosis, rheumatology

## Abstract

Vector-borne infections may underlie some rheumatic diseases, particularly in people with joint effusions. This study aimed to compare serum and synovial fluid antibodies to *B. burgdorferi* and *Bartonella* spp. in patients with rheumatic diseases. This observational, cross-sectional study examined paired synovial fluid and serum specimens collected from 110 patients with joint effusion between October 2017 and January 2022. Testing for antibodies to *B. burgdorferi* (using CDC criteria) and *Bartonella* spp. *via* two indirect fluorescent antibody (IFA) assays was performed as part of routine patient care at the Institute for Specialized Medicine (San Diego, CA, USA). There were 30 participants (27%) with positive two-tier *B. burgdorferi* serology and 26 participants (24%) with IFA seroreactivity (≥1:256) to *B. henselae* and/or *B. quintana*. Both *B. burgdorferi* IgM and IgG were detected more frequently in synovial fluid than serum: 27% of patients were either IgM or IgG positive in synovial fluid, compared to 15.5% in serum (*P* = 0.048). Conversely, *B. henselae* and *B. quintana* antibodies were detected more frequently in serum than synovial fluid; overall only 2% of patients had positive IFA titers in synovial fluid, compared to 24% who had positive IFA titers in serum (*P* < 0.001). There were no significant associations between *B. burgdorferi* or *Bartonella* spp. seroreactivity with any of the clinical rheumatological diagnoses. This study provides preliminary support for the importance of synovial fluid antibody testing for documenting exposure to *B. burgdorferi* but not for documenting exposure to *Bartonella* spp.

**IMPORTANCE:**

This study focuses on diagnostic testing for two common vector-borne diseases in an affected patient population. In it, we provide data showing that antibodies to *B. burgdorferi*, but not *Bartonella* spp., are more commonly found in synovial fluid than serum of patients with joint effusion. Since Lyme arthritis is a common—and sometimes difficult to diagnose—rheumatic disease, improving diagnostic capabilities is of utmost importance. While our findings are certainly not definitive for changes to practice, they do suggest that synovial fluid could be a useful sample for the clinical diagnosis of Lyme disease, and future prospective studies evaluating this claim are warranted.

## INTRODUCTION

*Borrelia burgdorferi, Bartonella henselae,* and *Bartonella quintana* are vector-borne diseases that can cause joint or bone pathology in both humans and other animal species (1–3). *Borrelia burgdorferi*, transmitted by *Ixodes scapularis* ticks and other tick species in the *Ixodes* genus, causes Lyme arthritis, with symptoms including joint swelling and pain (2, 3). *Bartonella* spp. are transmitted by various arthropod vectors, depending on the respective reservoir host. In humans, bartonellosis has been associated with an expanding spectrum of symptoms, clinical signs, and pathology, recently including arthritis, severe bone and joint pain, vasculopathy, and various neurological and psychiatric presentations (4–8).

Environmental factors, including microbial pathogens, have been implicated in disrupting immune tolerance and inducing autoimmune diseases such as rheumatoid arthritis, Sjogren’s syndrome, and immune-mediated vasculitis (9). However, the role of infectious agents in the pathogenesis of many rheumatic diseases has been incompletely studied, potentially leading to the underdiagnosis of pathogen-related rheumatic diseases in humans and other animals (10, 11). For example, *B. henselae* and *B. quintana* can both cause polyarthritis in association with culture-negative, vegetative valvular endocarditis but testing for these pathogens as part of the diagnostic workup is rarely performed (11). Coinfection with, or exposure to, combinations of multiple pathogens further complicates efforts to attain a definitive diagnosis, as co-infecting pathogens can induce synergistic pathologic effects in the host and may result in complex and atypical clinical presentations (12). Seroreactivity to both *B. henselae* and *B. burgdorferi* is often concurrently documented in both human patients and sick animals (13–15). Due to prior associations of both *B. burgdorferi* and *Bartonella* spp. with joint and bone pathology, studies that concurrently investigate these two genera are needed to better understand the medical implications of single or co-infection with these pathogens in rheumatic conditions (13, 16).

There is conflicting information on the accuracy of routine diagnostic laboratory tests for detecting exposure to, or infection with, *B. burgdorferi* and *Bartonella* spp. While the Centers for Disease Control and Prevention (CDC) has recommended two-tier testing for *B. burgdorferi* antibodies to diagnose Lyme disease—using first enzyme immunoassay (EIA) or immunofluorescence assay (IF) and then subsequent Western Blot for IgM and IgG on EIA or IFA-positive samples—this approach can lack sensitivity for the diagnosis of early *B. burgdorferi* infections, leading to false-negative results and underdiagnosis of Lyme disease (17). Also, serology cannot distinguish between active and inactive *B. burgdorferi* infections.

Some studies challenge the specificity of *B. burgdorferi* antibodies and their association with active infection, which is further confounded due to cross-reactivity with other spirochete bacteria leading to high false-positive rates (18). This cross-reactivity can lead to misdiagnoses of Lyme and delay the proper treatment of the disease (18). In addition, IgM antibodies may still be present and detectable despite the resolution of active infection (18). These high false-positive rates are seen with the ordering of clinical laboratory testing where testing is not indicated (18, 19).

Diagnostic assays for *Bartonella* spp. detection in patients suspected of bartonellosis also have varying clinical accuracy. Isolation of *Bartonella* spp., which is considered the gold standard for diagnosis, requires a long incubation period and is rarely successful when testing non-reservoir host species and immunocompetent patients (20, 21). Many approaches have been developed to improve *Bartonella* detection methods such as various types of PCR protocols, and DNA amplification equipment and enrichment cultures of blood, cerebrospinal fluid, joint fluid, and pathological effusions. The use of tissue samples, obtained from affected organs, may improve molecular detection of *Bartonella* spp. DNA, but there remain drawbacks and limitations to each assay (20, 22–24). Serology testing poses many issues as well. There is low specificity with ELISA due to IgG cross-reactivity between *Bartonella* species and *Coxiella burnetii*, and low sensitivity with IgM detection for *B. henselae* due to the geographic distribution of *Bartonella* species (25). Using indirect fluorescent antibody testing, a recent study failed to identify serologic cross-reactivity to *Coxiella burnetii* and several other zoonotic bacterial genera (26).

The use of synovial fluid to detect antibodies to *B. burgdorferi* has been debated in recent years. Some studies suggest that it may serve as a useful diagnostic tool, while others show that it can lead to the misdiagnosis of Lyme arthritis (27, 28). Conventional medical wisdom suggests that *Borrelia* and *Bartonella* antibodies cannot be detected in synovial fluid, and therefore serum antibody testing was thought to be the best method to detect exposure to *Borrelia burgdorferi* and *Bartonella* spp. However, clinicians’ experience has shown that antibodies can be detected in synovial fluid in the presence of joint pathologies. While one study has suggested that *B. henselae* humoral antibodies cannot be detected in synovial fluid, this study did not provide insight into other species in the genus such as *B. quintana* (29). These contradicting observations emphasize the need to investigate the utility of synovial fluid samples for improving the detection of exposure to these cryptic infections.

The goal of this study was to investigate exposure to *B. burgdorferi* and *Bartonella* spp. in patients with rheumatic disease, particularly focusing on the use of synovial fluid as a diagnostic specimen. The primary aim of this study was to compare the prevalence of *B. burgdorferi* and *Bartonella* spp. antibodies in serum versus synovial fluid in patients with rheumatic symptoms. We hypothesized that when compared to serum, synovial fluid would be more sensitive for the detection of antibodies to *B. burgdorferi,* but less sensitive for the detection of *Bartonella* spp. antibodies due to the propensities of *B. burgdorferi* to create predominantly joint pathologies and *Bartonella* spp. to create vascular pathologies. A secondary aim was to determine whether exposure to either pathogen was associated with clinical diagnoses; our null hypothesis was that there would be no associations with any specific clinical diagnosis because of the multifactorial and sometimes nebulous etiologies of these diagnoses.

## MATERIALS AND METHODS

### Setting and study design

This was an observational, cross-sectional study that was performed between October 2017 and January 2022 to determine exposure to *B. burgdorferi, B. henselae,* and *B. quintana* in people presenting for rheumatic conditions to the Institute for Specialized Medicine, San Diego, CA.

### Participants and study size

Patients who were having blood drawn and arthrocentesis performed as a component of their diagnostic procedures and had ultrasound-confirmed synovial effusions in large-sized or medium-sized joints, with calculated synovial effusion volume equal to or above 2 cubic centimeters per joint were asked to participate in this study. Participants were included if they provided a signed voluntary informed consent and there was an adequate amount of their serum and synovial fluid specimens remaining for research testing (100 μL or more for each sample type) after routine diagnostic tests were performed. This investigation was a clinical study using a convenience sample selected from the first approximately 100 consecutive patients who fit enrollment criteria.

At the time of initiation of this study, in October 2017, the following protocols were put in place by study authors to ensure compliance with the pre-2018 version of the Federal Policy for the Protection of Human Subjects: written informed consent was obtained from all study participants in accordance with federal regulations at the time, study participation involved minimal risk in that blood and synovial fluid samples were obtained only from people having blood and synovial fluid sampled for diagnostic purposes in the course of their medical care, all records were anonymized and no identifiable medical information was retained for research purposes. Investigational-use-only test results (antibody testing of synovial fluid) were used for research purposes only and results were not returned to study participants.

The study size was determined by the caseload at the study site, with enrollment conducted over a defined time period (10-26-2017 through 01-20-2022). For the 10 most common diagnoses (assuming that approximately half of the participants had the diagnosis and half did not), with 110 participants, there was an 80% power at alpha = 0.05 to detect a difference in pathogen exposure if approximately 10% of participants without the diagnosis had exposure compared to 35% of participants with the diagnosis. For less common diagnoses (assuming that 15% of participants had the diagnosis), with the same parameters, there was 80% power to detect a statistically significant difference if approximately 45% of participants with the diagnosis had exposure. People were classified by whether they had or did not have each of these specific diagnoses. Each included participant had at least one, if not more, diagnosis.

### Data sources and variables

#### Synovial fluid and serum sample collection

Synovial fluid was obtained *via* arthrocentesis performed under direct ultrasound guidance. Topical anesthesia was performed using ethyl chloride spray, then local anesthesia was administered by infiltrating soft tissues with 1% lidocaine. Blood samples were collected using a standard venipuncture technique. The collected samples of serum and synovial fluid were processed in accordance with the standard protocol and transported to Galaxy Laboratories, Research Triangle Park, NC, USA for *B. burgdorferi* and *Bartonella* spp. testing.

#### *B. burgdorferi* serology

*B. burgdorferi* exposure was determined using Clinical Laboratory Improvement Amendments (CLIA) validated, Commission on Office Laboratory Accreditation (COLA) accredited first-tier IgM and IgG ELISA and second-tier IgM and IgG Western blot testing, interpreted according to CDC criteria, as previously described (30 31). Both the first-tier ELISA and second-tier Western blot assays were run against whole cell derivatives of *Borrelia burgdorferi* B31 (32, 33). A serum or synovial fluid sample was considered *B. burgdorferi* seroreactive if the two-tier test (ELISA and western blot) result was positive for IgM, IgG, or both.

#### *Bartonella* spp. serology

CLIA-validated, COLA-accredited *B. henselae* and *B. quintana* IgG immunofluorescence antibody assay (IF) serology was performed as previously described (34). Cell culture-grown *B. henselae* SA2 and *B. quintana* were used as antigens. The reported titers for *B. henselae* and *B. quintana* IgG IFA ranged from <1:32 to ≥1:256 and are based upon a serial twofold dilution series of each human serum and synovial fluid sample. To further distinguish reactive from non-reactive sera, the laboratory grades fluorescence from 1 + to 4 + reactivity to ensure that fluorescence decreases incrementally as the dilution of patient serum and synovial fluid increases. Samples positive at ≥1:256 can be further tested to endpoint titer at the request of the ordering provider. For this study and to limit the potential for misinterpretation of non-specific binding, especially in a patient population with autoimmune disease, serum and synovial fluid samples were considered *B. henselae* or *B. quintana* seroreactive if the indirect fluorescent assay (IFA) resulted in an IgG titer ≥1:256. Antibody titers of 1:64 or 1:128 were considered indeterminate and titers of less than 1:64 were considered non-seroreactive.

Synovial fluid samples containing visible particulate matter were briefly centrifuged to pellet the material, and the resulting particulate-free material was used in sample analysis. Long-term synovial fluid optimization experiments (conducted between October 2017 and December 2018) were performed by assessment of serial dilutions of synovial fluid samples in assay buffer (1:25, 1:50, 1:100, and 1:200 in PBS-T for ELISA, 1:25 and 1:50 in TBS-T for western blot). Based on the results obtained from the optimization experiments, the 1:25 dilution was selected for IgM assays and 1:50 for IgG assays.

#### Clinical diagnoses

Clinical diagnoses were determined by the attending physician (AS). The diagnoses of interest used for the secondary aim were determined by evaluating the most common diagnoses in the samples. Some participants were diagnosed with more than one condition. If a participant had multiple common diagnoses, both diagnoses were included in the analysis. Some diagnoses of interest were combined into a more general category as follows: diagnoses of uterine cancer, prostate cancer, and thyroid carcinoma were categorized as “cancer”; any diagnoses that included specific neuropathy such as idiopathic peripheral neuropathy, recurrent median neuropathy, and chronic inflammatory demyelinating polyneuropathy (CIDP) were categorized as “neuropathy”; and diagnoses of both rheumatoid arthritis and juvenile rheumatoid arthritis were categorized as “rheumatoid arthritis.”

#### Laboratory assays

Although the article by Yang et al., 1992 utilized mouse serum and the article by Dressler et al., 1993 is not open access (but available at most University libraries or can be requested through University inter-library loan systems), the ELISA and western blot methods detailed in those references were those used by the authors for the development of the laboratory-developed assays utilized for these studies. Galaxy Diagnostics ELISA and western blot assays are CLIA-validated for human serum (accuracy and sensitivity including comparison to standard of care CDC Lyme Reference Panel I samples (35), inter- and intra-operator precision, and analytical specificity including cross-reactivity assessments) and have been commercially available since 2017. Per CLIA guidelines, these assays undergo CAP (College of American Pathologists)-administered proficiency testing twice per year where operators run blinded samples provided by CAP side-by-side with their regular clinical samples, which are propagated and prepared on slides in-house. The results are graded by CAP and provided to the laboratory. Assay operators also undergo yearly competency assessments, two components of which include running blinded samples and obtaining a >80% grade on a written technical assessment.

### Bias

Blood and synovial fluid specimens were drawn from a single physician’s practice in a single geographic location during the course of routine medical diagnosis and treatment, which may limit the generalizability of the results. Confounding effects of gender and age were accounted for in statistical analysis (see below).

### Statistical methods

All analysis was done using R v.4.2.2 (36). Descriptive statistics were calculated for demographic variables, serology results, and diagnoses of interest. Two-by-two tables were created and chi-squared tests were used to compare the proportion of people considered seroreactive using serum compared to synovial fluid. Differences between *B. burgdorferi* and *Bartonella* spp. seroreactive and non-seroreactive people for each clinical diagnosis were calculated using chi-squared or Fisher’s exact tests.

For the two most common clinical diagnoses of interest, multivariate logistic regression was used to determine the association between the diagnosis and pathogen exposure, including age and gender as potentially confounding covariates. We created four models. To investigate associations with osteoarthritis, we created two models with the dependent variable of clinical diagnosis of osteoarthritis (vs no diagnosis of osteoarthritis): one included *Bartonella* spp. exposure as an explanatory variable and one included *B. burgdorferi* exposure as an explanatory variable. We also created two models with the dependent variable of clinical diagnosis of Sjogren’s Syndrome (vs no diagnosis of Sjogren’s Syndrome): one included *Bartonella* spp. exposure as an explanatory variable and one included *B. burgdorferi* exposure as an explanatory variable. For all models, the *Bartonella* spp. exposure variable was a binary variable, with a positive defined as IFA seroreactivity against either *B. quintana* or *B. henselae* on serum or joint fluid. The *B. burgdorferi* exposure variable was a binary variable, with a positive defined as a two-tier positive for either IgG or IgM on either joint fluid or serum. For all models, age and gender were included: age as a continuous numerical variable using reported age in years, and gender as a categorical variable using reported gender (male or female). Odds ratios (ORs) and 95% confidence intervals (95% CIs) were calculated. The ORs for associations between OA or Sjogren’s and pathogen exposure, estimated from the multivariate logistic regression models, are adjusted (aOR). All other ORs are based on univariate models and therefore not adjusted.

A *P*-value of ≤0.05 was used to define statistical significance throughout the study. Though multiple comparisons were made for certain outcomes (e.g., odds of OA diagnosis evaluated with two logistic regression models), this was a retrospective cross-sectional study with small sample size, and the authors felt that using a more conservative *P*-value would not be useful in this context. The conclusions drawn from this study are hypothesis generating, and in that respect, the authors prioritize associations that may be biologically plausible for future prospective studies.

## RESULTS

### Participant demographics, sampling, and diagnoses

The study included 110 unique participants. Of those, 83 participants were sampled once, 15 participants were sampled twice, 7 participants were sampled three times, and 5 participants were sampled more than three times throughout the study period. For analyses, only the first sample from each unique participant was included (subsequent samples were excluded); the included samples were obtained from 2017-10-26 through 2022-01-20.

**Fig 1:**
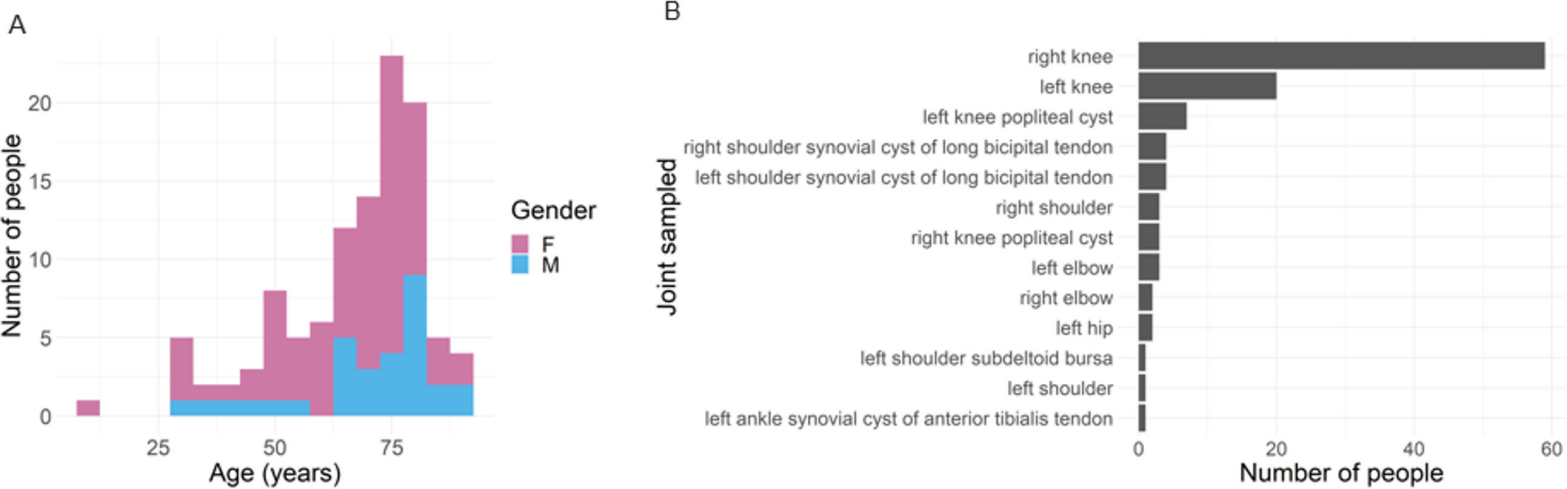
Study demographics. (**A**) Age and gender of participants. (**B**) Joint sampled for each study participant.

Of the 110 participants, 79 participants identified as female (72%), and 31 participants identified as male (28%; Fig. 1A). Participants ranged from 10 to 90 years of age, with a median age of 71 years (Fig. 1A). Joint fluid was collected from 13 different joints, with the right knee being the most frequently sampled, followed by the left knee and then left knee popliteal cysts (Fig. 1B). The joint sample location was related to the presenting problem of the participants and was included to fully describe the sample population but was not further analyzed in this study.

The 10 most frequent diagnoses were considered diagnoses of interest: osteoarthritis, Sjogren’s Syndrome, rheumatoid arthritis, chronic fatigue syndrome, gout, calcium pyrophosphate deposition, yersiniosis, cancer, rotator cuff disease, and neuropathy. Some participants were diagnosed with more than one condition and all diagnoses were included in the analysis. However, diagnoses of multiple conditions were not controlled for in the multivariate analysis. In all, 10 participants had no diagnoses of interest; 33 participants had 1 diagnosis of interest; 43 participants had two diagnoses of interest; 19 participants had three diagnoses of interest; and 5 participants had 4 diagnoses of interest. No participants had more than four diagnoses of interest. There were also 59 other diagnoses listed for participants that were categorized as “other,” and not listed as diagnoses of interest. Of the diagnoses of interests, osteoarthritis (OA) was the most common clinical diagnosis (67/110, 60.9%), followed by Sjogren’s Syndrome (42/110, 38.2%), rheumatoid arthritis (RA) (20/110, 18.2%), and chronic fatigue syndrome (CFS, 18/110, 16.4%); other diagnoses including gout, calcium pyrophosphate deposition, yersiniosis, cancer, neuropathy, and rotator cuff (RC) issues were each diagnosed in less than 16 participants (<15%).

### *B. burgdorferi* and *Bartonella* spp. seroreactivity in serum and synovial fluid

**Fig 2:**
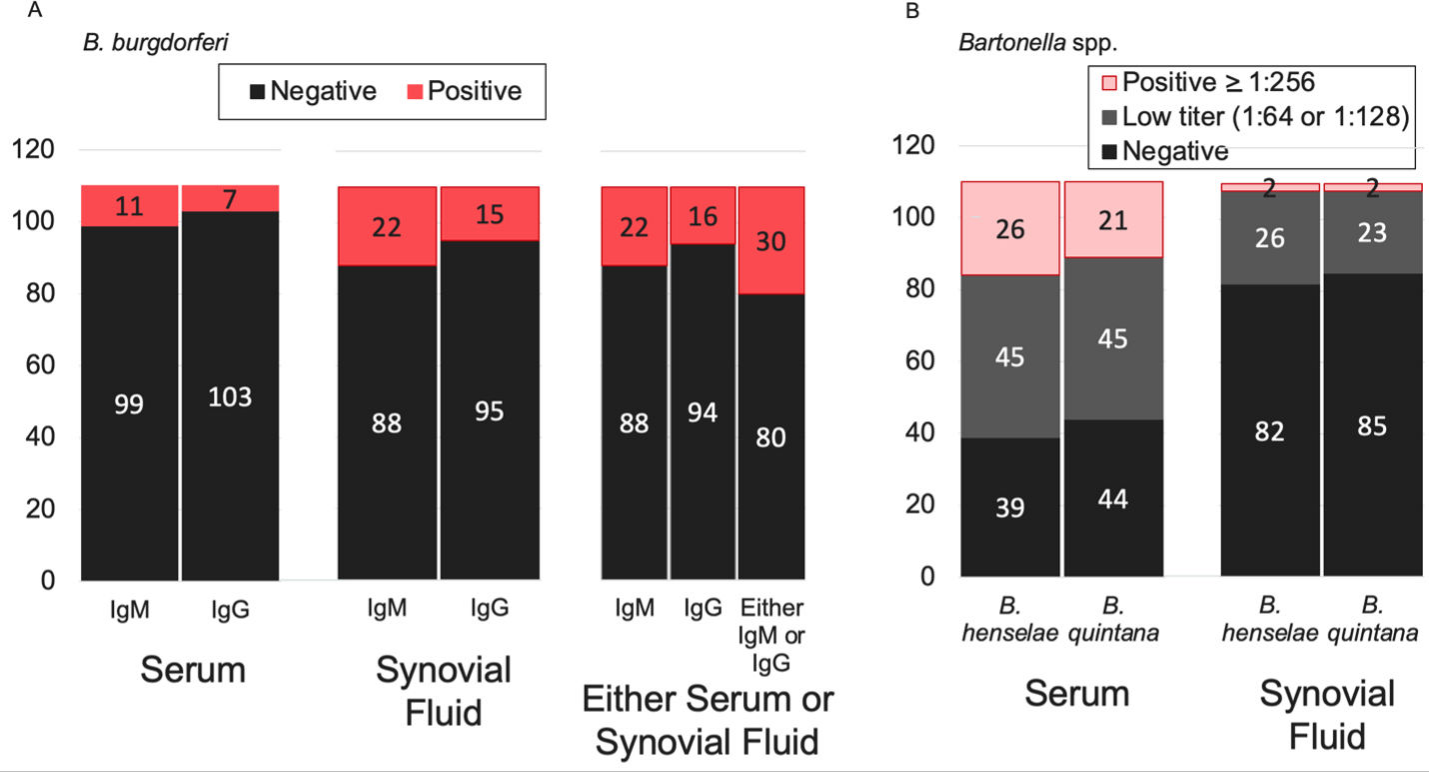
Serology results comparing serum to synovial fluid samples. (**A**) *B. burgdorferi* two-tier testing results, showing IgM and IgG. (**B**) *Bartonella* spp. IFA results, showing *B. henselae* and *B. quintana*.

When considering serum or synovial fluid seroreactivity, 30 participants (27%) were *B. burgdorferi* two-tier seroreactive for IgM, IgG, or both (Fig. 2A); 26 participants (24%) were *Bartonella* spp. seroreactive (Fig. 2B).

For *B. burgdorferi*, based on testing serum samples, there were 17 participants (15%) with evidence of *B. burgdorferi* exposure (Fig. 2A): 10 participants had IgM-positive, IgG-negative *B. burgdorferi* serology results, and 6 participants had IgM-negative, IgG-positive *B. burgdorferi* serology results. There was one participant with both *B. burgdorferi* IgM and IgG serum-positive results. Based on testing synovial fluid samples, there were 30 participants (27%) with evidence of *B. burgdorferi* exposure: 15 participants had IgM-positive, IgG-negative *B. burgdorferi* serology results, and 8 participants had IgM-negative, IgG-positive *B. burgdorferi* serology results. There were seven participants with both *B. burgdorferi* IgM- and IgG-positive synovial fluid results.

For *Bartonella* spp., 21 participants were seroreactive to both *Bartonella* spp. (19%, Fig. 2B); all participants that were *B. quintana* seroreactive were also *B. henselae* seroreactive. Only one participant was *B. henselae* seroreactive but not *B. quintana* seroreactive (four participants were *B. henselae* seroreactive and indeterminate for *B. quintana*).

Based on the serology results of *B. burgdorferi* between serum and synovial fluid samples for IgM and IgG, 11 participants were seroreactive for *B. burgdorferi* IgM in both serum and synovial fluid (Table 1); 6 participants were seroreactive *to B. burgdorferi* IgG in both serum and synovial fluid (Table 1); 17 participants were seroreactive for *B. burgdorferi* IgM and IgG in both serum and synovial fluid (Table 1). When comparing *B. burgdorferi* antibody detection in synovial fluid and serum, IgM and IgG were both detected more frequently in synovial fluid than in serum samples. For IgM, 11 more participants were positive on synovial fluid than on serum (Table 1). For IgG, eight more participants were positive on synovial fluid than on serum. (Table 1). One participant tested positive for *B. burgdorferi* in serum but not in synovial fluid. Combining IgM and IgG results, 27% of participants were antibody positive using synovial fluid, compared to only 15% using serum (*P* = 0.048) (Table 1).

**TABLE 1:** Comparison of results for *B. burgdorferi* two-tier serology on serum and synovial fluid for *B. burgdorferi* IgM, *B. burgdorferi* IgG, and *B. burgdorferi* IgM and IgG combined

	Synovial fluid Negative	Synovial fluid Positive
*B. burgdorferi* IgM		
Serum negative	88	11
Serum positive	0	11
*B. burgdorferi* IgG		
Serum negative	94	9
Serum positive	1	6
*B. burgdorferi IgM* and IgG		
Serum negative	80	13
Serum positive	0	17

Based on the serology results of *Bartonella* spp. between serum and synovial fluid samples, two participants were seroreactive for *B. henselae* in both serum and synovial fluid (Table 2); five participants were seronegative to *B. quintana* in synovial fluid but seroreactive to it in serum (Table 2). When *B. henselae* and *B. quintana* IFA in serum and synovial fluid were compared, 21 participants were positive for both *Bartonella* spp. (Table 3). When comparing *Bartonella* spp. antibody detection in synovial fluid and serum, antibodies to both *B. henselae* and *B. quintana* were detected more frequently in serum than in synovial fluid samples (Table 2). For both *B. henselae* and *B. quintana,* there were only two synovial fluid samples that were positive; both participants also were positive on serum samples.

**TABLE 2:** Comparison of results for *Bartonella* spp. IFA serology between serum and synovial fluid in *B. henselae* and *B. quintana*^*a*^

	Synovial fluid Negative	Synovial fluid Indeterminate	Synovial fluid Positive
*B. henselae*			
Serum negative	38	1	0
Serum indeterminate	37	8	0
Serum positive	7	17	2
*B. quintana*			
Serum negative	43	1	0
Serum indeterminate	37	7	0
Serum positive	5	15	2

^*a*^ Indeterminate includes IFA titers 1:64 or 1:128, positive includes IFA titers ≥ 1:256.

**TABLE 3:** Comparison of results for *Bartonella* spp. IFA serology between *B. henselae* and *B. quintana*^*a*^

	*B. quintana* IFA negative	*B. quintana* IFA indeterminate (1:64 or 1:128)	*B. quintana* IFA positive (≥1:256)
*B. henselae* IFA negative	17	2	0
*B. henselae* IFA indeterminate (1:64 or 1:128)	6	39	0
*B. henselae* IFA positive (≥1:256)	1	4	21

^*a*^ Indeterminate includes IFA titers 1:64 or 1:128, positive includes IFA titers ≥ 1:256.

Seroreactivity to both *B. burgdorferi* and *Bartonella* spp. together was uncommon: there were only nine participants (8%) seroreactive to both *Bartonella* spp. and *B. burgdorferi* (Table 4). In comparison, there were 21 participants with positive *B. burgdorferi* titers (on serum and/or synovial fluid) but negative for *Bartonella* spp., and 17 participants were *Bartonella* spp. IFA seroreactive, but negative for *B. burgdorferi* (Table 4). In all, 63 participants (57%) were seronegative for either pathogen (Table 4).

**TABLE 4:** Comparison of co-exposure between *Bartonella* spp. IFA serology (serum and synovial fluid) and *B. burgdorferi* two-tier serology (serum and synovial fluid)

	Co-exposure	*B. burgdorferi*	
		Negative	Positive
*Bartonella* spp.	Negative	63	21
	Positive	17	9

There were no statistically significant associations between age or gender and *B. burgdorferi* exposure (either IgM or IgG) (Table 5). There was no statistically significant association between age and *Bartonella* spp. exposure; however, *Bartonella* spp. exposure was more common in men (13/31, 42%) than women (13/79, 16%, *P* = 0.01) (Table 5).

**TABLE 5:** Associations between age or gender and *B. burgdorferi* exposure (either IgM or IgG), and *Bartonella* spp. exposure, with *P* < 0.05 indicating a statistically significant difference

	Negative	Positive	*P*-value
*B. burgdorferi* IgG or IgM result on serum or synovial fluid			
Age (median (range)), years	70.5 (31–90)	73.5 (10–90)	0.16
Number female, number male (% female)	57 F, 23M (71% female)	22 F, 8 M (73% female)	1
Bartonella spp. result on serum or synovial fluid (high titer)			
Age (median (range)), years	67.5 (10–90)	73.5 (30–90)	0.06
Number female, number male (% female)	66 F, 18 M (79% female)	13 F, 13 M (50% female)	0.01

### Association of *B. burgdorferi* and *bartonella* spp. on diagnoses of interest

OA, the most common diagnosis of interest, was reported in 67 participants (61%). Based on the multivariate logistic regression model (Table 6), the odds of an OA diagnosis increased with increasing age but were not significantly associated with gender. Controlling for age and gender, OA was not independently significantly associated with *B. burgdorferi* or *Bartonella* spp. seroreactivity. Sjogren’s syndrome, the second most common diagnosis of interest, was reported in 42 participants (38%). Based on the multivariate logistic regression model (Table 6), Sjogren’s syndrome was significantly less common in men compared to women but was not significantly associated with age. Controlling for age and gender, Sjogren’s syndrome was not independently significantly associated with *B. burgdorferi* or *Bartonella* spp. seroreactivity.

**TABLE 6:** Multivariate models for two most common diagnoses of interest: osteoarthritis and Sjogren’s syndrome^*a*^

	Osteoarthritis			Sjogren’s syndrome		
	*P*-value	OR	95% CI	*P*-value	OR	95% CI
*Bartonella* spp. multivariate model						
Age (years)	**<0.001**	**1.08**	**1.04–1.12**	0.27	0.99	0.96–1.01
Gender (male)	0.18	0.50	0.18–1.36	**0.003**	**0.18**	**0.05–0.52**
*Bartonella* spp. IFA (positive)	0.80	0.87	0.30–2.59	0.15	2.17	0.78–6.44
*B. burgdorferi* multivariate model						
Age (years)	**<0.001**	**1.08**	**1.04–1.12**	0.28	0.99	0.96–1.01
Gender (male)	0.14	0.47	0.17–1.26	**0.006**	**0.23**	**0.07–0.61**
*B. burgdorferi* seroreactive	0.31	0.59	0.21–1.65	0.94	1.03	0.41–2.55

^*a*^ Bold indicates a statistically significant independent association, with *P* < 0.05 indicating a statistically significant difference.

**Fig 3:**
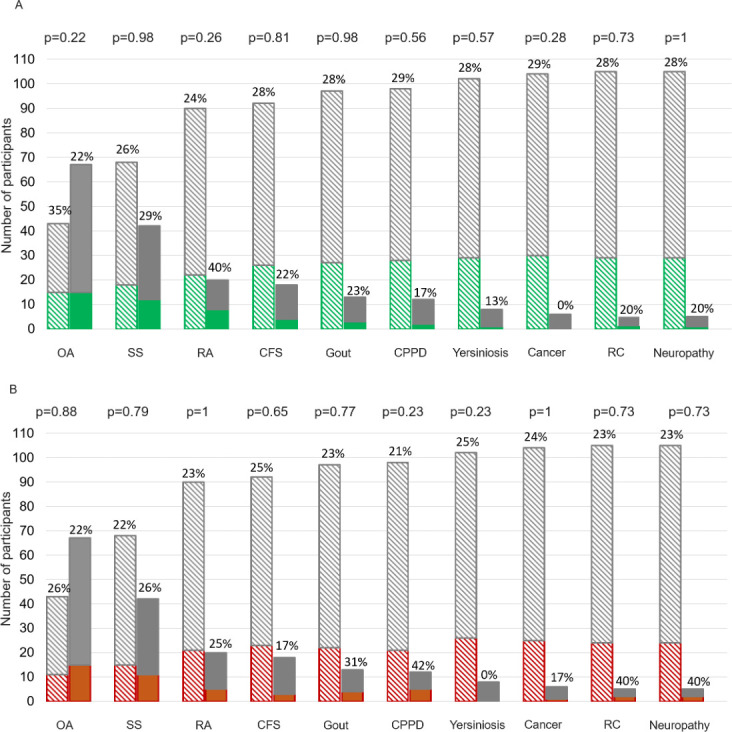
Percentage of participants with each diagnosis of interest and exposure to each pathogen. Solid bars indicate participants who did have the diagnosis, striped bars indicate participants without the diagnosis. Abbreviations: OA = osteoarthritis, SS = Sjogren’s syndrome, RA = rheumatoid arthritis, CFS = chronic fatigue syndrome, CPPD = calcium pyrophosphate deposition, RC = rotator cuff disease. A *P*-value of ≤0.05 has been used to indicate statistical significance between pathogens and diagnoses of interest. (**A**) *B. burgdorferi* exposure. Green indicates the proportion of participants with positive two-tier testing for IgM, IgG, or both (on serum and/or synovial fluid). (**B**) *Bartonella* spp. exposure. Orange indicates the proportion of participants with high titer seroreactivity to *B. henselae*, *B. quintana*, or both on serum (with or without antibodies detected in synovial fluid).

Figure 3 shows the proportion of participants with and without antibodies to each pathogen for each diagnosis of interest. There were no significant univariate associations between clinical diagnoses of interest and either *B. burgdorferi* or *Bartonella* spp. exposure (as defined by any one or more antibody test positive from either serum or synovial fluid).

## DISCUSSION

In this study, we compared serology results on paired serum and synovial fluid, using ELISA and WB (CDC two-tier criteria) for the detection of antibodies against *B. burgdorferi,* and IFA for the detection of antibodies against *B. henselae* and *B. quintana*. For *B. burgdorferi*, both IgM and IgG antibodies were significantly more commonly detected in synovial fluid than in serum (*P* = 0.048) (Table 1). The opposite was found for *Bartonella* spp., with only two participants positive on synovial fluid—both of whom had high serum antibody titers. These results suggest that testing synovial fluid for *B. burgdorferi* antibodies may provide more sensitive detection than testing serum alone. By contrast, testing synovial fluid for antibodies against *B. henselae* and *B. quintana* is unlikely to provide diagnostically relevant information for patients. Importantly, future studies should compare antibodies found in synovial fluid with direct detection methods and clinical outcomes.

A second finding of this study was a lack of statistically significant associations between *B. burgdorferi* and *Bartonella* spp. exposure with diagnoses of interest. The prevalence of many of these diagnoses—all but OA and Sjogren’s syndrome—was lower than needed in this study population, so the study lacked statistical power for evaluation of these less common diagnoses. Power was calculated based on a prevalence of 50%, which was only accurate for common diagnoses and not rare ones.

However, for OA and Sjogren’s syndrome, the study was adequately powered based on the power calculation outlined in the Materials and Methods section, and we were able to evaluate associations for these diagnoses while also controlling for age and gender as confounding demographic factors. In both cases, known associations with demographic factors were found: OA was associated with older age, and Sjogren’s syndrome was associated with the female gender. Even controlling for those factors, there was not a significant association between either of these diagnoses and *Bartonella* spp. or *B. burgdorferi* exposure in this study population. Based on our results, we did not find a statistically significant association between any one diagnosis and either pathogen. We were only powered to investigate OA and Sjogren’s syndrome due to the rarity of the other diagnoses of interest. Future studies should incorporate a more targeted sample population and better control studies to further investigate the association between exposure to these pathogens and rheumatic diseases because while our data were not statistically significant, they may be when mirrored in a larger population. There are many underlying explanations for these clinical diagnoses, and exposures were based on serology alone with no data on the duration of illness or previous treatments. For example, the high prevalence of cancer and OA in the population pool could be due to the median age being 71 years, and the predominance of women may also be related to sex differences in autoimmunity (37, 38). Many of these diagnoses of interest have multifactorial or incompletely understood etiologies, and therefore this study may not entirely capture whether or how these two pathogens affect the development of specific medical conditions. Identification of the mechanisms by which these infectious agents can cause diseases remains an important goal to provide efficient and optimal treatment to affected people.

Similar proportions of people in this study population were *B. burgdorferi* and *Bartonella* spp. seroreactive (27% and 24%, respectively). While the number of named or proposed *Bartonella* species now approaches 45, with some species, such as *B. henselae*, having a worldwide geographic distribution (10, 11, 39), *B. henselae* and *B. quintana* are currently considered the source of a majority of *Bartonella* infections in North America and Europe (40). Previous studies across various populations have shown that of patients with diagnosed Cat Scratch Disease (typically caused by *B. henselae* infection), about 10% developed musculoskeletal manifestations (41), 5% with atypical manifestations had neuritis or osteomyelitis (42), and about 3% had arthropathies (43). Conversely, of patients presenting for rheumatic conditions, anywhere from 0% to 60% have been reported to have evidence of *Bartonella* spp. infection or exposure (44–46).

The seroprevalence of *B. burgdorferi* and *Bartonella* spp. has been the subject of investigation many times but data remain varied and scarce. For example, the human seroprevalence of *B. burgdorferi* in California has been reported to be from 0.47% in a sample of 1700 blood donors (47) to 3.2% in a sample of 249 people from the public (48) to ≥24% in a sample of 83 people in a small rural community (49). The geographic distribution of *B. burgdorferi* infection also varies throughout the United States; Lyme disease was detected at significantly higher amounts in the upper Midwest, Northeast, and Mid-Atlantic regions, which accounted for 95.2% of all reported cases in the United States in a study that ranged from 2008 to 2015 (50). The seroprevalence of *Bartonella* spp. is also highly variable between geography and different demographic populations. For example, a study that investigated the seroprevalence of *Bartonella* spp. in the homeless population in California resulted in 10% for *B. quintana* and 4% for B. henselae (51) to as high as 28% in veterinary professionals and 23% in Californian foresters (52–54). Our study, which only involved patients evaluated by a rheumatologist, suggests a seroprevalence of 27% and 24% of *B. burgdorferi* and *Bartonella* spp. respectively, which is on the higher end of previously reported studies. The prevalence of both *B. burgdorferi* and *Bartonella* spp. in both humans and animals is expected to increase as climate change and global warming expands the geographic range of *B. burgdorferi* and *Bartonella* vectors (55).

Co-infection with and seroreactivity to multiple *Bartonella* species are not uncommon in humans. Rheumatic symptoms, including arthritis, have been reported in patients previously diagnosed with cat scratch disease, as well as patients with chronic bartonellosis (4, 56). However, as a subset of patients diagnosed with bartonellosis have also tested positive for *B. burgdorferi*, differentiating the relative contribution of these two pathogens to a patient’s rheumatic symptoms is difficult (4). Therefore, our data do not allow assessment of co-exposure to *B. burgdorferi* and *Bartonella* spp., which may have contributed to the development of our diagnoses of interests. Although *B. henselae* and *B. quintana* are considered the most common *Bartonella* species causing human infection in the United States, their relationship, as well as the relationship of other *Bartonella* spp. to which humans are exposed, with rheumatic symptoms needs to be further examined (55).

The zoonotic potential of both *B. burgdorferi* and *Bartonella* spp. make them pathogens of interest in human and veterinary medicine, as both can be transmitted from vector arthropods to humans, pets, and other animals. Currently, these infections are underreported, at least in part due to limitations of diagnostic testing modalities. For example, the Center for Disease Control and Prevention estimates that new Lyme Disease cases per year are underreported by a factor of 10, and in Canada, it is estimated that only 10% of Lyme disease cases are confirmed by serological testing and thus reported (57, 58). Currently, there are no national surveillance requirements for *Bartonella* spp. exposures. *Bartonella* spp. infection also suffers from underreporting because of vague clinical symptoms such as fever, weakness, headache, and joint pain, as well as its occasionally self-limiting nature (59). New strategies for serodiagnosis of both vector-borne diseases are needed; based on the results of this study, it may be reasonable to consider submitting synovial fluid to augment *B. burgdorferi* serologic testing, but there seems to be little added value in submitting synovial fluid for *Bartonella* spp. IFA serology.

Limitations of this study included the cross-sectional design and a lack of information about participants’ previous treatments targeted at *B. burgdorferi* and/or *Bartonella* spp. Other than osteoarthritis and Sjogren’s syndrome, there were low observation numbers for most diagnoses of interest. A larger prospective sample pool drawn from populations with specific diagnoses of interest (rather than *any* patient with joint effusion) and a robust control group would better allow us to see trends that were potentially obscured. In the future, a cohort study of healthy people with vector-borne disease exposure would be able to better determine the causality and directionality of pathogen exposure and diagnoses of interest because of the ability to follow participants over a period of time to determine the incidence of rheumatic manifestations. Some of this long-term epidemiologic work is already being addressed, such as studies describing long-term symptoms for patients diagnosed with acute Cat Scratch Disease in Israel, or the Study of Lyme Disease Immunology and Clinical Events (SLICE) study from Johns Hopkins University that follows patients after diagnosis of Lyme disease to examine risk factors, symptom severity, and immunologic biomarkers in Lyme disease patients over time (60, 61).

Despite these limitations, this study suggests that synovial fluid samples may be important in determining exposure to *B. burgdorferi* in patients with rheumatic symptoms*,* though may not provide additional information on exposure to *B. henselae* or *B. quintana* compared to serum alone. For *B. burgdorferi* antibodies, both IgM and IgG were more commonly detected in synovial fluid than in serum and often in synovial fluid only; in contrast, for *Bartonella* spp. antibodies were rarely detected in synovial fluid and never without their concurrent presence in serum. Therefore, clinicians should consider augmenting *B. burgdorferi* serodiagnosis with synovial fluid sampling and future studies should evaluate the use of synovial fluid for Lyme disease diagnosis. Future well-designed epidemiologic studies are needed to determine associations and possible causality and directionality between these pathogens and rheumatic manifestations.

## Data Availability

Raw data used for this study can be found through DataDryad (https://doi.org/10.5061/dryad.xwdbrv1mg) and are also available by request. To request this data, please contact Alexander Shikhman.
